# Longitudinal trends and determinants of dietary supplement administration during the first two years of life: a Korean birth cohort

**DOI:** 10.3389/fped.2026.1807460

**Published:** 2026-05-26

**Authors:** Jiyoung Jeong, Sukyung Kim, Minyoung Jung, Suk-Joo Choi, Soo-Young Oh, Sarang Jeong, Yoon Jung Yang, Jihyun Kim, Kangmo Ahn

**Affiliations:** 1 Department of Clinical Nutrition, Graduate of Future Strategy Convergence, Dongduk Women’s University, Seoul, Republic of Korea; 2 Department of Pediatrics, Samsung Medical Center, Sungkyunkwan University School of Medicine, Seoul, Republic of Korea; 3 Department of Obstetrics and Gynecology, Samsung Medical Center, Sungkyunkwan University School of Medicine, Seoul, Republic of Korea; 4 Department of Food and Nutrition, Dongduk Women’s University, Seoul, Republic of Korea

**Keywords:** birth cohort, cesarean section, dietary supplements, feeding methods, infants, longitudinal study

## Abstract

**Objectives:**

Dietary supplement administration has steadily increased in Korea, but evidence on administration of dietary supplements to infants remains limited. This study investigated the longitudinal trends and determinants of supplement administration in infants from birth to 24 months of age.

**Methods:**

A longitudinal birth cohort study enrolled 137 infants born at ≥28 weeks of gestation, and followed them at 2, 6, 12, and 24 months. Structured questionnaires assessed supplement administration, feeding methods, family history of allergic diseases, and demographics. Feeding was categorized as breastfeeding, formula feeding, or mixed feeding. Atopic dermatitis and food allergy were diagnosed using standardized criteria.

**Results:**

Overall, 82% of infants were given supplements during the study period. Probiotics and vitamin D were the most frequently administered at all ages, while multivitamin administration increased markedly after 12–24 months (38.6%). Supplement administration differed by feeding method: breastfed (29.4%) and mixed-fed (5.0%) infants had a higher percentage of iron supplementation at 12 months compared with formula-fed (0%). Multivitamin administration was more common among infants without a family history of allergies (low-risk group: 32.6%) compared with those with a family history of allergies (high-risk group: 23.4%). Infants born via cesarean section were significantly more likely to be given dietary supplements at 2–6 months (OR = 8.09, 95% CI: 1.79–36.49), 6–12 months (OR = 14.88, 95% CI: 1.91–116.10), and 12–24 months (OR = 5.16, 95% CI: 1.09–24.35).

**Conclusions:**

This study found a high percentage of supplement administration among Korean infants, with frequent administration of probiotics and vitamin D from early infancy. Over time, supplement types became more diverse, with usage trends varying by feeding method and allergy risk. Cesarean-born infants were more likely to be given supplements. These findings provide descriptive data on how supplements are administered to Korean infants and suggest a basis for exploring the implications of early-life supplement administration.

## 1 Introduction

The first 1,000 days, from conception to 24 months of age, are a critical period that significantly influences an infant's growth, development, and overall health. Nutritional intake during this period lays the foundation for lifelong health. Inadequate or excessive intake of nutrients during this period has been associated with developmental issues, such as impaired brain function, as well as increased risks of chronic diseases including obesity, type 2 diabetes, and hypertension (1–3).

Specifically, infants are particularly vulnerable to nutritional imbalances because their micronutrient requirements are remarkably high relative to their body weight due to rapid physical growth and neurodevelopment (4). While breast milk is the gold standard for infant nutrition and provides essential bioactive compounds, additional nutritional sources become important with growth and development. At the age of complementary feeding, nutrient requirements gradually begin to exceed the amounts provided by breast milk alone. In this context, dietary supplements may help address specific nutrient requirements that are difficult to meet through diet alone, even in the setting of a balanced diet (5). While dietary supplements are generally not considered essential for healthy children who consume a diverse and balanced diet (6), specific exceptions apply in infancy, when nutritional intake is primarily based on breast milk or formula and may not fully meet vitamin D requirements. Vitamin D is an essential nutrient for bone health and calcium metabolism during early infancy (7). Breast milk contains relatively low concentrations of vitamin D, which may be insufficient to maintain adequate vitamin D status without supplementation (7, 8). Sun exposure is also generally limited during early infancy, further supporting the importance of adequate vitamin D intake (7, 8). In accordance with international recommendations for rickets prevention, universal vitamin D supplementation (400 IU/day) is recommended for all infants beginning shortly after birth (9–11). There is no unified national guideline in Korea addressing routine nutritional supplementation in infants; instead, recommendations are distributed across dietary reference standards and consensus-based pediatric literature. The 2020 Dietary Reference Intakes for Koreans (KDRI), issued by the Ministry of Health and Welfare and the Korean Nutrition Society, recommend an adequate intake of 5 μg/day (approximately 200 IU) of vitamin D for infants, reflecting a population-based dietary standard rather than a clinical supplementation guideline (12). In contrast, pediatric subspecialty literature and society-endorsed reviews in Korea consistently support routine vitamin D supplementation of approximately 400 IU/day during infancy, largely based on international evidence and the high prevalence of vitamin D insufficiency reported in Korean children (7, 13).

Iron is a key micronutrient involved in physical growth, neurodevelopment, cellular function, and hormone synthesis (12, 14). Iron stores accumulated during gestation typically begin to deplete by 4 to 6 months of age, making breastfed infants susceptible to iron deficiency if complementary foods are inadequate (14). Based on recommendations from the American Academy of Pediatrics (AAP), iron supplementation (1 mg/kg/day of elemental iron) is recommended from approximately 4 months of age for exclusively breastfed infants (15). However, iron supplementation recommendations are not standardized at the national level in Korea. Current Korean pediatric practice follows a risk-based approach, recommending iron supplementation for exclusively breastfed infants and high-risk groups such as preterm or low-birth-weight infants, alongside the introduction of iron-rich complementary foods from 4 to 6 months of age (16). In this context, according to the KDRI, the recommended dietary allowance for iron is set at 6 mg/day for infants aged 6–11 months, reflecting increased physiological requirements during late infancy (12).

While vitamin D supplementation recommendations are relatively consistent across countries, iron supplementation guidelines vary considerably depending on regional and clinical contexts. In this regard, nutritional supplementation practices in Korea reflect a hybrid framework in which vitamin D supplementation is broadly recommended based on consensus-level evidence, whereas iron supplementation remains individualized in the absence of a standardized national policy.

Despite these clinical guidelines, data from the Korea National Health and Nutrition Examination Survey (KNHANES) indicate that among all children and adolescents in Korea, dietary supplement administration is most prevalent among toddlers aged 1–3 years (17). The types of supplements consumed have also shown changing trends that follow market trends. In a 2007–2009 survey, vitamin and mineral supplements were the most consumed type of supplement by children aged 2–6, followed by red ginseng, colostrum, and omega–3 fatty acids (18). Subsequently, in 2010–2012 and 2015–2017 surveys, probiotics emerged as the most consumed supplement, followed by vitamins and minerals (17, 19). These nationwide surveillance data play an important role in identifying nutritional trends and informing public health strategies for Korean children (13). These findings also suggest that the actual landscape of supplement administration may deviate from established clinical recommendations and may be increasingly influenced by commercial marketing and parental concerns regarding immune health.

Most previous studies have been limited to cross-sectional analyses, which restrict the ability to identify changes in consumption patterns over time and their associated factors (3, 5, 17–20). Therefore, this study utilizes birth cohort data to conduct a longitudinal analysis of dietary supplement administration up to 24 months of age. We aim to examine how supplement usage varies according to general characteristics, such as feeding method and allergy risk status, and to identify which supplements are predominantly administered, and at which specific time points.

## 2 Materials and methods

### 2.1 Study population and design

This prospective birth cohort study was conducted at Samsung Medical Center, Seoul, Korea. Pregnant women at ≥28 weeks of gestation were recruited between February 2017 and 2020. At enrollment, parents completed questionnaires on demographic characteristics and family history of allergic diseases and underwent skin prick tests to eight inhalant allergens. Based on allergy history and skin prick test results, infants were classified into allergy high-risk or control groups. Participants were excluded if they did not meet these eligibility criteria (e.g., sensitization without clinical allergic disease) or declined participation after screening (Supplementary Figure 1). Baseline infant clinical characteristics and dietary supplement administration during the first 2 months of life were assessed at the 2-month follow-up visit, which served as the baseline timepoint for postnatal analyses. Infants were followed at 2, 6, 12, and 24 months of age, and 137 infants who completed the follow-up questionnaires were included. This study was approved by the Institutional Review Board of Samsung Medical Center (IRB No. SMC-2016-12-111), and written informed consent was obtained from all parents prior to participation. The study protocol was registered in the World Health Organization International Clinical Trials Registry Platform (KCT0007979).

### 2.2 Demographic data collection

Information on infant sex, gestational age, mode of delivery, maternal age, maternal smoking status, parental education level, and household income was collected via a self-administered questionnaire. Postnatal anthropometric measurements were performed by trained medical staff during hospital visits using standardized clinical equipment. Body weight and recumbent length were measured using a pediatric anthropometric measurement system (GL-310, G-Tech International, Seoul, Korea), and measurements were recorded to the nearest 0.1 kg and 0.1 cm, respectively. Infants were measured without shoes and wearing light clothing and diapers. Recumbent length was measured up to 12 months of age, and standing height was measured at 24 months using a standard stadiometer (DS-B03, Jenix, Korea). Preterm birth was defined as delivery before 37 weeks of gestation. Smoking status was classified based on the question: “Have you ever smoked more than 100 cigarettes (5 packs) in your lifetime?” Participants who answered “No” were categorized as non-smokers, and those who answered “Yes” were categorized as smokers.

### 2.3 Data collection

A family history of allergic diseases was considered positive if one of the following criteria was met: (1) at least one parent had both a positive skin prick test and a history of asthma or allergic rhinitis, or (2) at least one parent or sibling had physician-diagnosed atopic dermatitis (AD). Infants were evaluated for AD and food allergy (FA) at each visit. AD was diagnosed according to the Hanifin and Rajka criteria (21). FA was confirmed by either a positive oral food challenge or a convincing history of immediate reactions within 2 h of food ingestion, together with a positive serum-specific IgE (≥0.35 kU/L, ImmunoCAP; Thermo Fisher Scientific, Waltham, MA, USA).

### 2.4 Dietary supplement and feeding method assessment

Dietary supplement administration was assessed through structured questionnaires completed by parents at each follow-up visit. Dietary supplements were defined as any orally administered products given to infants with the intention of supplementing the diet, including vitamins, minerals, probiotics, and other commercially available health-related products. At the 2-month visit, parents were asked whether their infant had received vitamin D or probiotic supplements during the first 2 months of life (0–<2 months) (yes/no). For other dietary supplements, parents were asked to report any products given to their infants using open-ended responses. If supplement administration was reported, parents were asked to provide additional information including the product name (brand) and the period of administration (start and end dates). At the 6-, 12-, and 24-month visits, parents reported supplement administration during the interval since the previous visit. Reported products were reviewed by the research team and categorized into predefined supplement groups. The number of parents who completed the supplement-related questionnaires was 137 at 2 months, 132 at 6 months, 127 at 12 months, and 107 at 24 months. Feeding practices were assessed at each follow-up visit by asking parents, “What type of milk has your baby consumed (breast milk and/or formula)?” Based on the cumulative responses over the first 12 months, participants were classified into three groups: Breastfeeding (BF): infants who received only breast milk for the entire period; Formula-feeding (FF): infants who received only formula for the entire period; and Mixed-feeding (MF): infants who received both breast milk and formula at any point during the follow-up period.

### 2.5 Statistical analysis

Analyses were performed separately at each age interval (0–<2, 2–<6, 6–<12, and 12–<24 months) using available cases with non-missing data for the variables included in that specific analysis. No imputation was performed. Because the sample size was determined by the number of participants available during follow-up, no *a priori* power calculation was performed. The subgroup analyses should therefore be considered exploratory. As this was an exploratory study aiming to identify potential associations and avoid missing clinically relevant trends (Type II error), multiple testing corrections were not applied. Data were analyzed using SAS version 9.4 (SAS Institute Inc., Cary, NC, USA). The Mann–Whitney U test was used to compare continuous variables, which are presented as median and interquartile range (IQR). Categorical variables were compared using Fisher's exact test due to small sample sizes in certain subgroups. Descriptive statistics were used to present dietary supplement administration by age, feeding method, and family history of allergies. Logistic regression analysis was used to examine predictors of dietary supplement administration in infants. *P*-values less than 0.05 were considered statistically significant.

## 3 Results

### 3.1 General characteristics of infants according to dietary supplement administration

A total of 137 infants were included in the analysis. Their baseline characteristics are summarized in Table 1. Among them, 58.4% were male, 40.9% were delivered by cesarean section, and 68.6% had a family history of allergic diseases. Table 2 presents the general characteristics of infants according to dietary supplement administration. Overall, 69.3% of infants received supplements in early infancy, rising to 84.8% at 2 to <6 months, 86.6% at 6 to <12 months, and 86.9% at 12 to <24 months. From 2 to 24 months, supplement administration was consistently higher in infants born via cesarean section, than in those delivered vaginally (*p* = 0.002 at 2 to <6 months; *p* = 0.001 at 6 to <12 months; *p* = 0.039 at 12 to <24 months). At 12 to <24 months, supplement administration was more frequent among infants diagnosed with AD at 12 months (*p* = 0.039). There were no significant differences in birth weight, sex, gestational age, prematurity, timing of complementary food introduction, maternal smoking, or AD at 2 or 6 months, or FA at 12 months.

**Table 1:** Baseline characteristics of the study population.

Variable	Total (*n* = 137)
Birth weight (kg)	3.22 ± 0.41
Male sex	80 (58.4)
Gestational age (weeks)	39.10 ± 1.27
Cesarean section (yes)	56 (40.9)
Family history of allergic diseases (yes)	94 (68.6)
Maternal age (years)	33.42 ± 3.22
Maternal smoking (yes)	14 (10.2)
Maternal education	
High school	1 (0.7)
University	100 (73.0)
Graduate degree or higher	36 (26.3)
Paternal education	
High school	6 (4.4)
University	106 (77.4)
Graduate degree or higher	25 (18.2)
Household income	
<4,000,000 KRW	39 (28.5)
4,000,000–5,999,999 KRW	37 (27.0)
≥6,000,000 KRW	61 (44.5)

Values are presented as number (%) or mean ± standard deviation.

**Table 2:** General characteristics of infants according to dietary supplement administration.

Characteristics	0-<2 mo			2-<6 mo			6-<12 mo			12-<24 mo		
	Supplement administered (*n* = 95)	Non administered (*n* = 42)	*p* value^a^	Supplement administered (*n* = 112)	Non administered (*n* = 20)	*p* value	Supplement administered (*n* = 110)	Non administered (*n* = 17)	*p* value	Supplement administered (*n* = 93)	Non administered (*n* = 14)	*p* value
Birth weight (kg)	3.2 (3.0–3.5)	3.3 (3.0–3.4)	0.994	3.2 (3.0–3.5)	3.3 (3.0–3.4)	0.936	3.2 (3.0–3.5)	3.3 (3.0–3.4)	0.781	3.3 (3.0–3.5)	3.2 (3.0–3.4)	0.535
Sex			0.059			1.000			0.605			0.394
Male	50 (52.6)	30 (71.4)		66 (58.9)	12 (60.0)		66 (60.0)	9 (52.9)		58 (62.4)	7 (50.0)	
Female	45 (47.4)	12 (28.6)		46 (41.1)	8 (40.0)		44 (40.0)	8 (47.1)		35 (37.6)	7 (50.0)	
Gestational age at birth (weeks)	39.2 (38.3–40.0)	39.4 (38.4–40.2)	0.442	39.2 (38.3–40.1)	39.6 (38.4–40.1)	0.570	39.2 (38.3–40.0)	39.6 (38.3–40.2)	0.990	39.3 (38.4–40.0)	39.5 (38.0–40.0)	0.996
Premature infant (<37 weeks)			1.000			0.594			0.183			0.434
Yes	7 (7.4)	0 (0.0)		7 (6.3)	0 (0.0)		4 (3.6)	2 (11.8)		3 (3.2)	1 (7.1)	
No	88 (92.6)	42 (100.0)		105 (93.8)	20 (100.0)		106 (96.5)	15 (88.2)		90 (96.8)	13 (92.9)	
Birth mode			0.132			0.002			0.001			0.039
Vaginal delivery	51 (54.3)	29 (69.1)		59 (52.7)	18 (90.0)		57 (51.8)	16 (94.1)		50 (53.8)	12 (85.7)	
Cesarean section	43 (45.7)	13 (31.0)		53 (47.3)	2 (10.0)		53 (48.2)	1 (5.9)		43 (46.2)	2 (14.3)	
Introduction of complementary food			0.156			0.193			0.472			0.747
≥4 mon	17 (18.3)	14 (34.2)		23 (20.5)	8 (40.0)		23 (21.1)	6 (35.3)		22 (24.2)	3 (21.4)	
≥5 mon	55 (59.1)	20 (48.8)		65 (58.0)	9 (45.0)		61 (56.0)	8 (47.1)		47 (51.7)	9 (64.3)	
≥6 mon	21 (22.6)	7 (17.1)		24 (21.4)	3 (15.0)		25 (22.9)	3 (17.7)		22 (24.2)	2 (14.3)	
Maternal smoking			0.226			0.694			1.000			0.593
Yes	12 (12.6)	2 (4.8)		13 (11.6)	1 (5.0)		11 (10.0)	1 (5.9)		8 (8.6)	0 (0.0)	
No	83 (87.4)	40 (95.2)		99 (88.4)	19 (95.0)		99 (90.0)	16 (94.1)		85 (91.4)	14 (100)	
Atopic dermatitis at 2mon			0.701			1.000			1.000			1.000
Yes	5 (5.3)	3 (7.1)		6 (5.4)	1 (5.0)		6 (5.5)	0 (0.0)		5 (5.4)	0 (0.0)	
No	90 (94.7)	39 (92.9)		106 (94.6)	19 (95.0)		104 (94.6)	17 (100)		88 (94.6)	5 (100)	
Atopic dermatitis at 6mon			0.613			0.745			0.307			0.119
Yes	14 (14.9)	8 (19.5)		18 (16.1)	4 (20.0)		19 (17.3)	1 (5.9)		17 (18.5)	0 (0.0)	
No	80 (85.1)	33 (80.5)		94 (83.9)	16 (80.0)		91 (82.7)	16 (94.1)		75 (81.5)	14 (100)	
Atopic dermatitis at 12mon			0.235			0.364			0.521			0.039
Yes	15 (17.1)	11 (27.5)		20 (18.9)	6 (30.0)		23 (21.1)	2 (11.8)		22 (23.9)	0 (0.0)	
No	73 (83.0)	29 (72.5)		86 (81.1)	14 (70.0)		86 (78.9)	15 (88.2)		70 (76.1)	14 (100)	
Food allergy at 12mon			0.742			0.686			0.643			0.353
Yes	7 (8.0)	4 (9.8)		9 (8.5)	2 (10.0)		9 (8.26)	2 (11.8)		10 (10.8)	0 (0.0)	
No	81 (92.1)	37 (90.2)		97 (91.5)	18 (90.0)		100 (91.7)	15 (88.2)		83 (89.3)	14 (100)	

Values are presented as number (%) or median (interquartile range).

a *P*-values were calculated using the Mann–Whitney U test for continuous variables. Categorical variables were compared using Fisher's exact test due to the small sample sizes in certain subgroups.

### 3.2 Dietary supplement administration among infants

**Figure 1:**
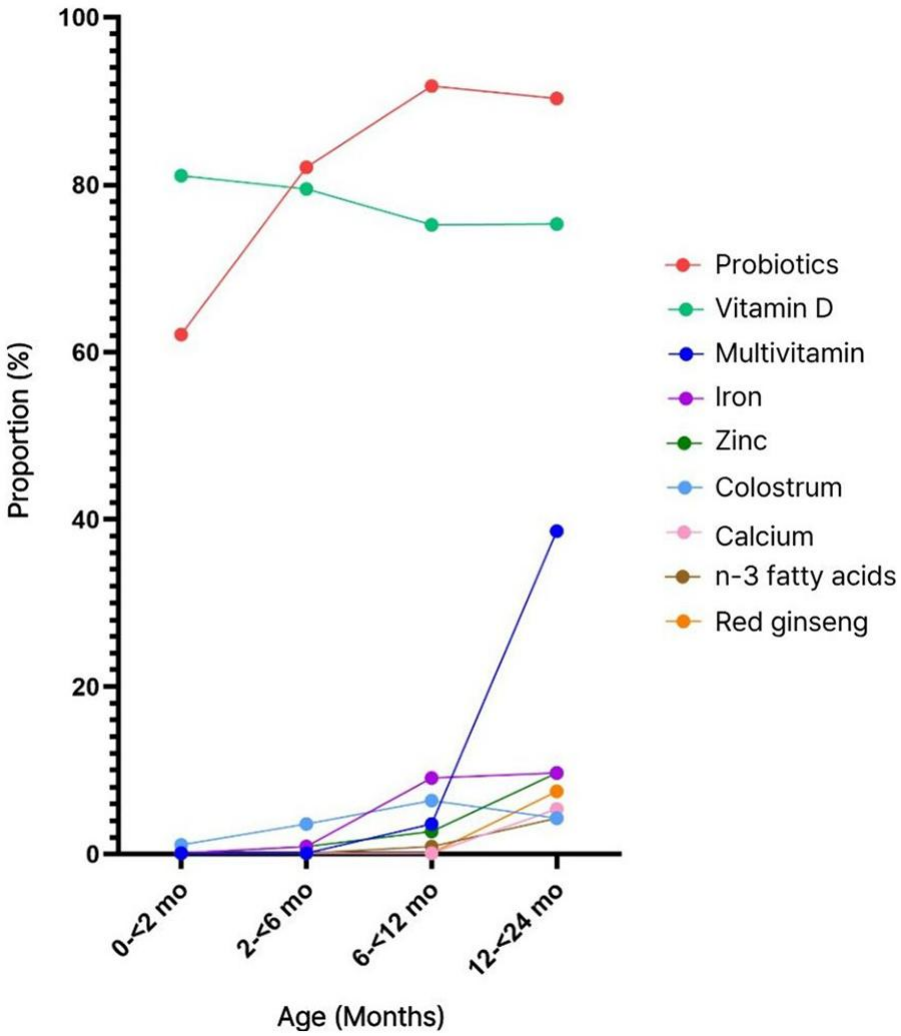
Supplement administration trends in infants aged 0–24 months.

Table 3 summarizes the percentage of dietary supplement administration among infants from birth to 24 months. Figure 1 presents the overall trends in supplement use during this period. In addition, Supplementary Figure 2 illustrates the trajectories of supplement use across infancy, highlighting changes and transitions in usage behaviors over time. Probiotic and vitamin D supplements were the most frequently given across all age groups. At 0 to <2 months, 62.1% of infants received probiotic supplements, and 81.1% received vitamin D supplements. The intake of probiotics increased with age, reaching 91.8% at 6 to <12 months, and 90.3% at 12 to <24 months. Vitamin D intake remained consistently high throughout infancy, ranging from 75.2% to 81.1%. From 6 months of age, the variety of supplements administered increased. Multivitamin supplementation was initiated in later infancy, with 3.6% of infants receiving it at 6 to <12 months, and 38.6% at 12 to <24 months. At 12 to <24 months, the most commonly administered supplements, in descending order: probiotics, vitamin D, multivitamins, iron, zinc, red ginseng, calcium, n–3 fatty acids, colostrum, magnesium, propolis, and vitamin C. Intake of iron and zinc supplements also increased with age, reaching 9.7% at 12 to <24 months for both. Colostrum supplement administration peaked at 6 to <12 months (6.4%). Other supplements, such as calcium, n–3 fatty acids, red ginseng, magnesium, propolis, and vitamin C were given to 0.9% to 7.5% of infants.

**Table 3:** Dietary supplement administration in infants from birth to 24 months of age.

	0-<2 mo		2-<6 mo		6-<12mo		12-<24mo	
	(*n* = 95)		(*n* = 112)		(*n* = 110)		(*n* = 93)	
	*n*	%	*n*	%	*n*	%	*n*	%
Probiotic	59	62.1	92	82.1	101	91.8	84	90.3
Vitamin D	77	81.1	89	79.5	82	75.2	70	75.3
Multivitamin	0	0.0	0	0.0	4	3.6	36	38.6
Iron	0	0.0	1	0.9	10	9.1	9	9.7
Zinc	0	0.0	1	0.9	3	2.7	9	9.7
Colostrum	1	1.1	4	3.6	7	6.4	4	4.3
Calcium	0	0.0	0	0.0	1	0.9	5	5.4
n-3 fatty acids	0	0.0	0	0.0	1	0.9	5	5.4
Red ginseng	0	0.0	0	0.0	0	0.0	7	7.5
Magnesium	0	0.0	0	0.0	1	0.9	3	3.2
Propolis	0	0.0	0	0.0	0	0.0	2	2.2
Vitamin C	0	0.0	0	0.0	1	0.9	1	1.1

### 3.3 Dietary supplement administration in infants by feeding method

 Table 4 presents the percentage of dietary supplement administration according to feeding method. At 0 to <2 months, the administration of probiotic supplements was highest in the FF group (54.6%), followed by the MF group (45.8%) and the BF group (25.9%). Vitamin D supplementation was comparable between the MF group (59.4%) and the BF group (59.3%), while notably lower in the FF group (18.2%). At 2 to <6 months, probiotic intake increased across all feeding groups, with the highest percentage observed in the MF group (73.3%), followed by the FF group (71.8%) and the BF group (62.1%). Vitamin D supplement administration was highest in the BF group (82.8%), followed by the MF group (70.0%) and the FF group (53.9%). The intake of colostrum supplements remained low overall, but was highest in the FF group (7.7%). At 6 to <12 months, probiotic intake remained high across all groups: 82.5% in the MF group, 75.0% in the FF group, and 64.7% in the BF group. Similarly, vitamin D supplementation was most prevalent in the MF group (67.7%), followed by the BF group (70.6%) and the FF group (33.3%). The intake of multivitamins, iron, zinc, colostrum, calcium, n–3 fatty acids, magnesium, and vitamin C were generally low, and primarily observed in the FF and MF groups.

**Table 4:** Dietary supplement administration in infants by feeding method.

	BF		FF		MF	
	*n*	%	*n*	%	*n*	%
0-<2 mo						
n	27		11		96	
Probiotic	7	25.9	6	54.6	44	45.8
Vitamin D	16	59.3	2	18.2	57	59.4
Colostrum	0	0.0	0	0.0	1	1.0
2-<6 mo						
n	27		11		96	
Probiotic	18	62.1	28	71.8	44	73.3
Vitamin D	24	82.8	21	53.9	42	70.0
Colostrum	0	0.0	3	7.7	1	1.6
Iron	0	0.0	0	0.0	1	1.6
Zinc	0	0.0	0	0.0	1	1.6
6-<12 mo						
n	25		76		29	
Probiotic	11	64.7	9	75.0	80	82.5
Vitamin D	12	70.6	4	33.3	65	67.7
Multivitamin	1	5.9	1	8.3	2	2.0
Iron	5	29.4	0	0.0	5	5.0
Zinc	1	5.9	0	0.0	2	2.0
Colostrum	0	0.0	1	8.3	6	5.9
Calcium	0	0.0	1	8.3	0	0.0
n-3 fatty acids	0	0.0	1	8.3	0	0.0
Magnesium	0	0.0	1	8.3	0	0.0
Vitamin C	0	0.0	1	8.3	0	0.0

BF, breastfeeding; FF, formula feeding; MF, mixed feeding.

### 3.4 Dietary supplement administration in infants by family history of allergies

The percentage of dietary supplement administration according to family history of allergies is presented in Table 5. At 0 to <2 months, probiotic intake was higher in the high-risk group (47.9%) compared to the low-risk group (32.6%), while vitamin D intake was similar between groups (55.3% vs. 58.1%). At 2 to <6 months, probiotic and vitamin D intake increased, reaching comparable levels in both groups (approximately 69%–70%). Colostrum, iron, and zinc supplements were given at low rates, primarily in the high-risk group. At 6 to <12 months, probiotic intake remained high in both groups (79.1%, 80.5%), and vitamin D supplementation was more frequent in the high-risk group (68.2%). Intake of colostrum, iron, and zinc was slightly higher in the low-risk group. Multivitamins and other supplements were given to 1.1% to 4.3% of high-risk infants. At 12 to <24 months, probiotic and vitamin D intake remained high and similar between groups. Multivitamin intake was more common in the low-risk group (32.6%) than in the high-risk group (23.4%). Iron and zinc intake also showed a slightly higher proportion in the low-risk group. Other supplements were given to 1.1% to 7.0% of infants

**Table 5:** Dietary supplement administration in infants by family history of allergies.

	High-risk group		Low-risk group	
	(*n* = 94)		(*n* = 43)	
	*n*	%	*n*	%
0-<2 mo				
Probiotic	45	47.9	14	32.6
Vitamin D	52	55.3	25	58.1
Colostrum	0	0.0	1	2.3
2-<6 mo				
Probiotic	62	69.7	30	69.8
Vitamin D	59	66.3	30	69.8
Colostrum	2	2.1	2	4.7
Iron	1	1.1	0	0.0
Zinc	1	1.1	0	0.0
6-<12 mo				
Probiotic	68	79.1	33	80.5
Vitamin D	58	68.2	24	58.5
Colostrum	3	3.2	4	9.3
Iron	6	6.4	4	9.3
Zinc	2	2.1	1	2.3
Multivitamin	4	4.3	0	0.0
Vitamin C	1	1.1	0	0.0
Magnesium	1	1.1	0	0.0
Calcium	1	1.1	0	0.0
n-3 fatty acids	1	1.1	0	0.0
12-<24 mo				
Probiotic	56	77.8	28	80.0
Vitamin D	47	65.3	23	65.7
Colostrum	3	3.2	1	2.3
Iron	5	5.3	4	9.3
Zinc	6	6.4	3	7.0
Multivitamin	22	23.4	14	32.6
Vitamin C	1	1.1	0	0.0
Magnesium	3	3.2	0	0.0
Calcium	3	3.2	2	4.7
n-3 fatty acids	3	3.2	2	4.7
Red ginseng	4	4.3	3	7.0
Propolis	2	2.1	0	0.0

### 3.5 Predictors of dietary supplement administration in infants

Univariable logistic regression analyses identified predictors of dietary supplement administration among infants from birth to 24 months of age (Table 6). Male infants were less likely than female infants to receive supplements at 0 to <2 months (OR = 0.44, 95% CI: 0.20–0.97). Delivery by cesarean section was significantly associated with an increased likelihood of receiving supplement compared to vaginal delivery at 2 to <6 months (OR = 8.09, 95% CI: 1.79–36.49), 6 to <12 months (OR = 14.88, 95% CI: 1.91–116.10), and 12 to <24 months (OR = 5.16, 95% CI: 1.09–24.35). AD at 24 months was inversely associated with supplement administration at 0 to <2 months (OR = 0.39, 95% CI: 0.15–0.99). Higher maternal age was positively associated with supplement administration at 6 to <12 months (OR = 1.21, 95% CI: 1.02–1.43). No statistically significant associations were observed with birth weight, birth order, gestational age, parental education, maternal smoking, or household income in any age group.

**Table 6:** Predictors of dietary supplement administration in infants.

Variable	0-<2 mo		2-<6 mo		6-<12 mo		12-<24 mo	
	OR (95% CI)	*p* value	OR (95% CI)	*p* value	OR (95% CI)	*p* value	OR (95% CI)	*p* value
Infant characteristics								
Birth weight (kg)	1.10 (0.44–2.73)	0.846	0.93 (0.29–3.02)	0.905	0.97 (0.28–3.42)	0.967	1.12 (0.27–4.66)	0.878
Birth order (≥2 vs first-born)	1.92 (0.50–7.34)	0.342	0.93 (0.18–4.66)	0.926	0.73 (0.14–3.77)	0.708	0.56 (0.10–3.04)	0.500
Male sex (vs female)	0.44 (0.20–0.97)	0.042	0.96 (0.36–2.53)	0.929	1.33 (0.48–3.72)	0.583	1.66 (0.54–5.12)	0.380
Gestational age at birth (weeks)	0.88 (0.65–1.19)	0.400	0.90 (0.61–1.33)	0.605	1.00 (0.67–1.49)	0.990	1.09 (0.70–1.70)	0.700
Cesarean section (vs vaginal delivery)	1.88 (0.87–4.06)	0.108	8.09 (1.79–36.49)	0.007	14.88 (1.91–116.10)	0.010	5.16 (1.09–24.35)	0.038
Introduction of complementary food (Reference category: ≥4 mon)								
≥ 5 mon	2.27 (0.95–5.42)	0.343	2.51 (0.87–7.28)	0.424	1.99 (0.62–6.36)	0.576	0.71 (0.18–2.89)	0.368
≥ 6 mon	2.47 (0.81–7.50)	0.312	2.78 (0.66–11.80)	0.401	2.17 (0.49–9.72)	0.524	1.50 (0.23–9.87)	0.483
Atopic dermatitis at 24 months	0.39 (0.15–0.99)	0.047	0.42 (0.14–1.27)	0.125	0.96 (0.25–3.67)	0.946	1.56 (0.32–7.58)	0.580
Food allergy at 24 months	0.71 (0.25–2.00)	0.516	1.67 (0.35–7.93)	0.516	0.79 (0.20–3.07)	0.730	2.74 (0.33–22.43)	0.348
Parental characteristics								
Maternal age	0.97 (0.86–1.09)	0.582	1.15 (0.99–1.34)	0.077	1.21 (1.02–1.43)	0.028	1.15 (0.97–1.37)	0.116
Maternal smoking (vs non-smoking)	2.89 (0.62–13.54)	0.178	2.50 (0.31–20.21)	0.392	1.78 (0.22–14.72)	0.594	2.88 (0.13–62.41)	0.500
Maternal education Graduate degree or higher (vs University graduation)	0.72 (0.32–1.62)	0.429	1.01 (0.34–3.04)	0.983	1.69 (0.45–6.32)	0.434	1.37 (0.35–5.31)	0.651
Paternal education Graduate degree or higher (vs University graduation)	2.73 (0.88–8.54)	0.084	2.10 (0.45–9.76)	0.344	1.69 (0.36–7.97)	0.510	1.26 (0.26–6.20)	0.774
Monthly household income (Reference category: <4,000,000 KRW)								
4,000,000–5,999,999 KRW	1.23 (0.47–3.25)	0.817	0.81 (0.22–2.92)	0.819	0.73 (0.18–2.96)	0.813	1.29 (0.26–6.29)	0.574
≥ 6,000,000 KRW	1.24 (0.52–2.96)	0.761	0.83 (0.26–2.72)	0.878	0.70 (0.19–2.51)	0.701	0.76 (0.20–2.84)	0.482

OR, odds ratio; CI, confidence interval.

Odds ratios, 95% confidence intervals, and *p*-values were obtained from univariable logistic regression models.

Reference categories are indicated in parentheses.

## 4 Discussion

In this longitudinal birth cohort study, we observed a high percentage (82%) of dietary supplement administration in Korean infants from birth to 24 months of age, considerably higher than rates previously reported in community-based studies from Korea (39.2%), the United States (18.2%), and Australia (23.5%) (17, 22, 23). One plausible explanation is that although our cohort was designed as a general population study, many participants were recruited from families residing near the study hospital. This catchment area is characterized by relatively high socioeconomic status and strong parental concern for child health, which may have contributed to the elevated prevalence of supplement administration.

Current pediatric guidelines, including those from the AAP, recommend vitamin D supplementation of 400 IU/day for all infants beginning shortly after birth to prevent deficiency and support bone health (10, 11, 15, 16). Iron supplementation is also recommended from approximately 4 months of age for predominantly breastfed infants because breast milk alone may not provide sufficient iron to meet increasing requirements during later infancy (15, 16). In Korea, routine supplementation recommendations for healthy infants primarily focus on these nutrients, while the routine administration of other dietary supplements is not formally recommended. Consistent with these recommendations, vitamin D supplementation was commonly reported in our cohort. Notably, a recent systematic review reported that global adherence to vitamin D guidelines varies widely (20%–90%), with higher rates often observed during early infancy (24). Our findings of sustained high intake up to 24 months suggest that caregivers in this study area maintain an exceptionally high commitment to clinical guidelines, possibly due to elevated health literacy. Although dosage information was not available, the high prevalence suggests good adherence to current vitamin D supplementation recommendations.

From 6 to 12 months of age, the types of supplements administered became more diverse, with a marked increase in the intake of colostrum and iron supplements. Iron supplementation in particular was more common among infants who were breastfed or mixed-fed. This may be explained by the fact that while breast milk offers numerous immunological benefits, its iron content is low (approximately 0.35 mg/L), making it insufficient to meet the iron requirements of infants after six months of age (15). This pattern may reflect adherence to clinical recommendations encouraging iron supplementation among predominantly breastfed infants. These findings underscore the importance of reinforcing guideline-based iron supplementation practices, particularly among breastfed infants at risk of iron deficiency. Despite these recommendations, the relatively low rate of iron supplementation observed among breastfed infants in our cohort suggests a gap between clinical guidance and real-world practice. Further research is needed to better understand the factors influencing caregiver decision-making and adherence to iron supplementation recommendations.

Similarly, a 2025 nationwide study in Germany identified iron as a critical nutrient, noting that many infants do not meet the required intake levels through complementary foods alone (25). The essential nutrients considered critical for infants in Korea primarily include vitamin D, iron, calcium, DHA, and zinc (26, 27). These align with the types of nutrients currently being supplemented by the infants in this study. Previous research has reported that breastfed and mixed-fed infants had a significantly higher risk of iron-deficiency anemia, highlighting the necessity for iron supplementation in these groups (28). However, our data do not allow us to determine whether parents’ supplementation practices were influenced by clinical recommendations, or by their own awareness, so it remains unclear to what extent these practices reflect adherence to professional guidance. After 12 months of age, multivitamins use increased, with a higher percentage among infants in the low-risk allergy group, compared to those in the high-risk group. This suggests that caregivers of high-risk infants may be more cautious about the efficacy of multivitamins, or may be using them more selectively, based on recommendations from healthcare professionals.

Supplement intake was observed from the early stages of infancy, with vitamin D and probiotics being the first supplements introduced and consistently given up to 24 months of age, regardless of feeding method or family history of allergy. Notably, probiotic use showed an increasing trend from 62% at 0 to <2 months, to 82% at 2 to <6 months. This suggests an age-related increase in probiotic use during early infancy, consistent with previous reports describing early introduction of probiotics (17, 19, 29). We did not observe a clear association between antibiotic exposure and probiotic use in our cohort (data not shown). This pattern may instead reflect parental perceptions of benefits for infant gut and immune health, as well as increasing public interest and the widespread availability of probiotic products for infants (23). In contrast, probiotics were the most frequently administered supplement across all age groups despite the absence of guideline-based recommendations. This discrepancy may reflect parental perceptions of potential health benefits rather than evidence-based recommendations, highlighting the need for clearer guidance regarding appropriate supplement administration during infancy.

Feeding method may also explain some of the observed supplementation patterns. For example, iron supplementation was not observed among formula-fed infants in our cohort, which is clinically plausible because commercially available infant formulas are typically iron-fortified and provide sufficient iron intake (14, 30). Similarly, vitamin D supplementation was less frequently reported among formula-fed infants in early infancy, possibly because vitamin D-fortified formulas can provide adequate vitamin D intake when consumed in sufficient amounts (11).

Infants born via cesarean section were significantly more likely to be given dietary supplements, compared to those born vaginally. Vaginal delivery facilitates the transfer of beneficial bacteria and immune-related substances from the mother's birth canal to the infant, which positively influences the development of the early immune system. In contrast, cesarean delivery bypasses this exposure, leading to the establishment of microbial communities that resemble maternal skin flora (31–33). This may raise caregiver concerns regarding immune competence, potentially resulting in a greater reliance on dietary supplementation. Indeed, systematic reviews have demonstrated that supplementation with *Lactobacillus* and *Bifidobacterium* in cesarean-born infants can improve gut microbial composition and enhance immune function (34). Our findings illustrate how both biological considerations and caregiver perceptions converge to influence supplement administration in this subgroup.

This study has several limitations. First, the relatively small sample size limits the generalizability of the findings, and larger, population-based cohort studies are needed to confirm these results. Second, although designed as a general population cohort, the recruitment setting may have overrepresented families with higher socioeconomic status and strong health interest, which may limit generalizability. Third, multiple testing corrections were not performed in our analyses. While this approach was chosen to maintain the exploratory power of the study and avoid missing potentially important associations (Type II error), the results should be interpreted with caution regarding the risk of Type I error. Despite these limitations, this study holds significant value, as it provides a longitudinal follow-up across the critical first 24 months of life, standardized diagnostic criteria for allergic diseases confirmed by pediatric allergists, and high-quality data collection through face-to-face interviews by trained research personnel. To our knowledge, few studies in Korea have examined infant supplement administration using this level of temporal detail. The findings can serve as foundational data to develop evidence-based guidelines for supplement administration and nutrition policies targeting infants and young children.

In conclusion, dietary supplement administration was highly prevalent among Korean infants, with probiotics and vitamin D most frequently consumed from early infancy. The mode of delivery, sex, maternal age, and allergic disease risk were significantly associated with variations in supplement use over time. Infants born via cesarean section were more likely to be given supplements, possibly due to caregiver concerns about gut and immune health resulting from altered microbial colonization. These findings highlight the gap between clinical guideline recommendations and real-world practices, emphasizing the interplay of clinical needs, parental perceptions, and societal influences. A key strength of this study is its longitudinal tracking of temporal changes in supplement use throughout infancy, which provides foundational evidence to support the development of updated, evidence-based public health guidelines and caregiver education for safe supplement administration in early childhood.

## Data Availability

The datasets presented in this article are not publicly available due to privacy and ethical restrictions but may be available from the corresponding author upon reasonable request and with appropriate institutional approval.

## References

[1] Beluska-Turkan K Korczak R Hartell B Moskal K Maukonen J Alexander DE Nutritional gaps and supplementation in the first 1000 days. Nutrients. (2019) 11(12):2891. 10.3390/nu11122891 31783636 PMC6949907

[2] Koletzko B Brands B Poston L Godfrey K Demmelmair H. Early nutrition programming of long-term health. Proc Nutr Soc. (2012) 71(3):371–8. 10.1017/s0029665112000596 22703585

[3] Woźniak D Przysławski J Banaszak M Drzymała-Czyż S. Dietary supplements among children ages 0–3 years in Poland-are they necessary? Foods. (2022) 12(1):16. 10.3390/foods12010016 36613232 PMC9818416

[4] Schwarzenberg SJ Georgieff MK. Advocacy for improving nutrition in the first 1000 days to support childhood development and adult health. Pediatrics. (2018) 141(2):e20173716. 10.1542/peds.2017-3716 29358479

[5] Bailey RL Fulgoni VL 3rd Keast DR Dwyer JT. Dietary supplement use is associated with higher intakes of minerals from food sources. Am J Clin Nutr. (2011) 94(5):1376–81. 10.3945/ajcn.111.020289 21955646 PMC3192481

[6] Shaikh U Byrd RS Auinger P. Vitamin and mineral supplement use by children and adolescents in the 1999-2004 National Health and Nutrition Examination Survey: relationship with nutrition, food security, physical activity, and health care access. Arch Pediatr Adolesc Med. (2009) 163(2):150–7. 10.1001/archpediatrics.2008.523 19188647 PMC2996491

[7] Heo JS Ahn YM Kim AE Shin SM. Breastfeeding and vitamin D. Clin Exp Pediatr. (2022) 65(9):418–29. 10.3345/cep.2021.00444 34902960 PMC9441616

[8] Suksantilerd S Thawatchai R Rungrojjananon N. Prevalence of vitamin D deficiency in exclusively breastfed infants at Charoenkrung Pracharak Hospital. World J Clin Pediatr. (2024) 13(1):86693. 10.5409/wjcp.v13.i1.86693 38596439 PMC11000061

[9] Munns CF Shaw N Kiely M Specker BL Thacher TD Ozono K Global consensus recommendations on prevention and management of nutritional rickets. Horm Res Paediatr. (2016) 85(2):83–106. 10.1159/000443136 26741135

[10] Wagner CL Greer FR. American Academy of Pediatrics section on breastfeeding; American academy of pediatrics committee on nutrition. Prevention of rickets and vitamin D deficiency in infants, children, and adolescents. Pediatrics. (2008) 122(5):1142–52. 10.1542/peds.2008-1862 18977996

[11] Braegger C Campoy C Colomb V Decsi T Domellof M Fewtrell M Vitamin D in the healthy European paediatric population. J Pediatr Gastroenterol Nutr. (2013) 56(6):692–701. 10.1097/MPG.0b013e31828f3c05 23708639

[12] Ministry of Health and Welfare, The Korean Nutrition Society. Dietary reference intakes for Koreans 2020. Sejong: Ministry of Health and Welfare (2020).

[13] Kang M Choi SY Jung M. Dietary intake and nutritional status of Korean children and adolescents: a review of national survey data. Clin Exp Pediatr. (2021) 64(9):443–58. 10.3345/cep.2020.01655 33445834 PMC8426097

[14] Domellöf M Braegger C Campoy C Colomb V Decsi T Fewtrell M Iron requirements of infants and toddlers. J Pediatr Gastroenterol Nutr. (2014) 58(1):119–29. 10.1097/mpg.0000000000000206 24135983

[15] Baker RD Greer FR Co Nutrition. Diagnosis and prevention of iron deficiency and iron-deficiency anemia in infants and young children (0–3 years of age). Pediatrics. (2010) 126(5):1040–50. 10.1542/peds.2010-2576 20923825

[16] Ahn HSSH, editors. Hong Chang’s Pediatrics. 12th ed. Seoul: MiraeN (2022).

[17] Jeon JH Seo MY Kim SH Park MJ. Dietary supplement use in Korean children and adolescents, KNHANES 2015-2017. Public Health Nutr. (2021) 24(5):957–64. 10.1017/s1368980020003419 33040740 PMC10195559

[18] Yoon JY Park HA Kang JH Kim KW Hur YI Park JJ Prevalence of dietary supplement use in Korean children and adolescents: insights from Korea National Health and Nutrition Examination Survey 2007-2009. J Korean Med Sci. (2012) 27(5):512–7. 10.3346/jkms.2012.27.5.512 22563216 PMC3342542

[19] Kang DS Lee KS. The status of dietary supplements intake in korean preschool children: data from the Korea national health and nutrition examination survey 2010-2012. Pediatr Gastroenterol Hepatol Nutr. (2014) 17(3):178–85. 10.5223/pghn.2014.17.3.178 25349834 PMC4209323

[20] Bailey RL Gahche JJ Thomas PR Dwyer JT. Why US children use dietary supplements. Pediatr Res. (2013) 74(6):737–41. 10.1038/pr.2013.160 24002333 PMC3873849

[21] Hanifin JM Rajka G. Diagnostic features of atopic dermatitis. Acta Derm Venereol (Stockh). (1980) 60(92):44–7. 10.2340/00015555924447

[22] O’Brien SK Malacova E Sherriff JL Black LJ. The prevalence and predictors of dietary supplement use in the Australian population. Nutrients. (2017) 9(10):1154. 10.3390/nu9101154 29065492 PMC5691770

[23] Gahche JJ Herrick KA Potischman N Bailey RL Ahluwalia N Dwyer JT. Dietary supplement use among infants and toddlers aged< 24 months in the United States, NHANES 2007–2014. J Nutr. (2019) 149(2):314–22. 10.1093/jn/nxy269 30753556 PMC6551282

[24] Eslami O Cuskelly GJ O'Connor Á. Adherence to vitamin D supplementation guidelines in children under five years of age: a systematic literature review. Eur J Nutr. (2024) 63(1):79–92. 10.1007/s00394-023-03255-0 37792100

[25] Burgard L Spiegler C Jansen S Brettschneider AK Straßburg A Alexy U Critical vitamin D and iron intakes in infants aged 6–11 months: results from the nationwide German KiESEL study. Front Nutr. (2025) 12:1472685. 10.3389/fnut.2025.1472685 40034734 PMC11872716

[26] Jung JH Kim EA Lee SY Moon JE Lee EJ Park SH. Vitamin D Status and factors associated with vitamin D deficiency during the first year of life in preterm infants. Nutrients. (2021) 13(6):2019. 10.3390/nu13062019 34208333 PMC8231173

[27] Hong J Chang JY Shin S Oh S. Breastfeeding and red meat intake are associated with iron status in healthy Korean weaning-age infants. J Korean Med Sci. (2017) 32(6):974–84. 10.3346/jkms.2017.32.6.974 28480656 PMC5426231

[28] Clark KM Li M Zhu B Liang F Shao J Zhang Y Breastfeeding, mixed, or formula feeding at 9 months of age and the prevalence of iron deficiency and iron deficiency anemia in two cohorts of infants in China. J Pediatr. (2017) 181:56–61. 10.1016/j.jpeds.2016.10.041 27836288 PMC5274569

[29] Chen Y-C Chien Y-W Chang P-J Hsieh W-S Chen P-C. Probiotic supplement use among young children in Taiwan: a prospective cohort study. (2012)

[30] Fewtrell M Bronsky J Campoy C Domellöf M Embleton N Fidler Mis N Complementary feeding: a position paper by the European Society for Paediatric Gastroenterology, Hepatology, and Nutrition (ESPGHAN) Committee on Nutrition. J Pediatr Gastroenterol Nutr. (2017) 64(1):119–32. 10.1097/mpg.0000000000001454 28027215

[31] Dominguez-Bello MG Costello EK Contreras M Magris M Hidalgo G Fierer N Delivery mode shapes the acquisition and structure of the initial microbiota across multiple body habitats in newborns. Proc Natl Acad Sci U S A. (2010) 107(26):11971–5. 10.1073/pnas.1002601107 20566857 PMC2900693

[32] Neu J Rushing J. Cesarean versus vaginal delivery: long-term infant outcomes and the hygiene hypothesis. Clin Perinatol. (2011) 38(2):321–31. 10.1016/j.clp.2011.03.008 21645799 PMC3110651

[33] Chua WC Chen Y-L Yen C-F Chen H-L. Long-term health outcomes of children born by cesarean section: a nationwide population-based retrospective cohort study in Taiwan. J Formos Med Assoc. (2025) 124(11):1034–8. 10.1016/j.jfma.2024.09.024 39358115

[34] Inchingolo F Inchingolo AD Palumbo I Trilli I Guglielmo M Mancini A The impact of cesarean section delivery on intestinal Microbiota: mechanisms, consequences, and perspectives-a systematic review. Int J Mol Sci. (2024) 25(2):1055. 10.3390/ijms25021055 38256127 PMC10816971

