# Supplementary material for: Longitudinal trends and determinants of dietary supplement administration during the first two years of life: a Korean birth cohort
Source: Front Pediatr. 2026 May 26;14:1807460. doi: 10.3389/fped.2026.1807460 (PMC13246621; doi:10.3389/fped.2026.1807460)
Supplement: Supplementary file 1 [file Supplementaryfile1.docx]

Supplementary Material

# Supplementary figure

**
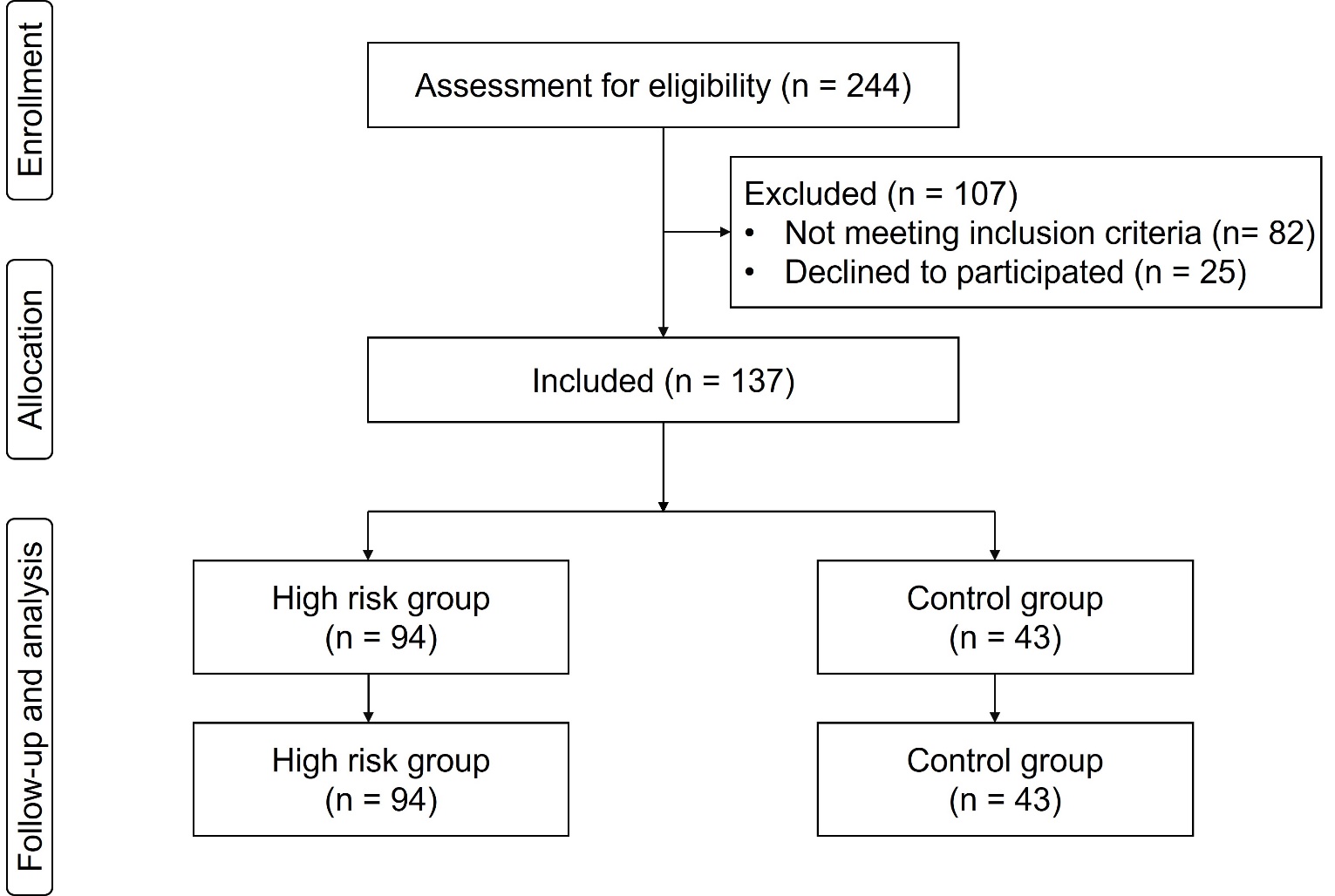
**

**Supplementary figure 1**. Flow diagram of the study population.


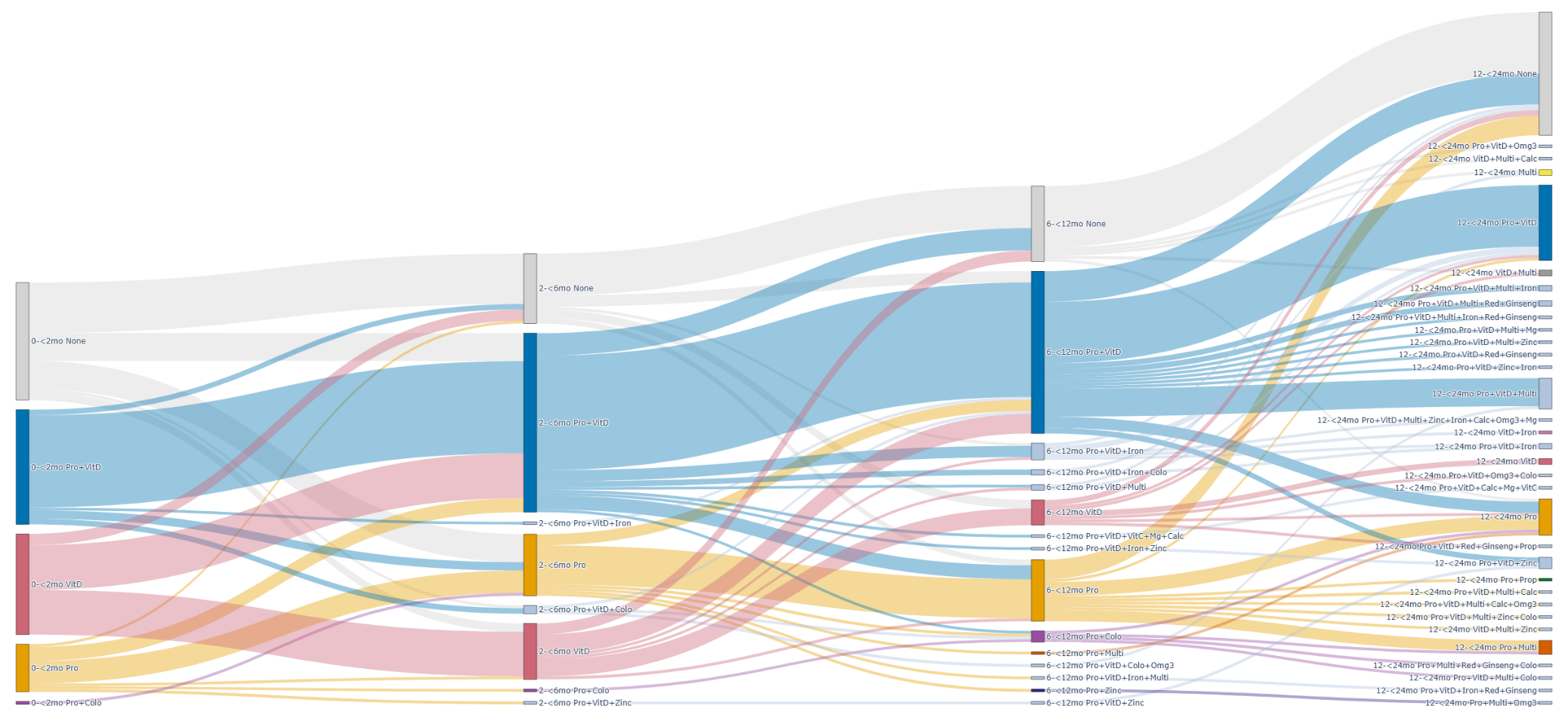


**Supplementary figure 2**. Patterns of dietary supplement use in infants aged 0–24 months

Abbreviation; Probiotics, Pro; Vitamin D, VitD; Multivitamin, Multi; Colostrum, Colo; Calcium, Calc; n-3 fatty acids, Omg3; Red ginseng, RGen; Propolis, Prop; Vitamin C, VitC; Magnesium, Mg
